# Impact of SARS-CoV-2 lockdown on expansion of HIV transmission clusters among key populations: A retrospective phylogenetic analysis

**DOI:** 10.1016/j.lana.2022.100369

**Published:** 2022-09-23

**Authors:** Rachel L. Miller, Angela McLaughlin, Vincent Montoya, Junine Toy, Sarah Stone, John Harding, Richard H. Liang, Jason Wong, Rolando Barrios, Julio S.G. Montaner, Jeffrey B. Joy

**Affiliations:** a British Columbia Centre for Excellence in HIV/AIDS, Vancouver, BC, Canada; b Bioinformatics Program, University of British Columbia, Vancouver, BC, Canada; c Vancouver Coastal Health, Vancouver, BC, Canada; d British Columbia Centre for Disease Control, Vancouver, BC, Canada; e Department of Medicine, University of British Columbia, Vancouver, BC, Canada

**Keywords:** HIV, SARS-CoV-2, COVID-19, Public health, Phylogenetics, Transmission clusters, MSM, PWID

## Abstract

**Background:**

Public health measures designed to reduce SARS-CoV-2 transmission led to reduced access to care and prevention services for people living with or at risk of acquiring HIV, particularly during the initial introduction of extensive restrictions. This reduction in access may have contributed to increases in HIV transmission not outweighed by decreases in transmission occurring as a result of reduced contact rates promoted by the same public health measures.

**Methods:**

We synthesize available province-wide HIV data in British Columbia, Canada, together with public mobility data to phylogenetically investigate the early impacts of SARS-CoV-2 on HIV transmission. Cluster growth, coalescent branching events and lineage-level diversification rates were assessed in “pre-lockdown” (January 22–March 21, 2020), “lockdown” (March 22–May 20, 2020) and “post-lockdown” (May 21–July 19, 2020) to facilitate comparison of transmission trends across key populations.

**Findings:**

Results reveal increased HIV transmission in a limited number of clusters in association with reduced access to health services during the initial introduction of SARS-CoV-2-related restrictions. In particular, clusters associated with people who inject drugs (PWID) show rapid growth, extensive branching events in phylogenetic trees during and following the lockdown period, and elevated median change in individuals’ viral diversification rates during lockdown compared to clusters associated with men who have sex with men (MSM), consistent with increased transmission rates between PWID.

**Interpretation:**

Increased vigilance and innovative targeted solutions are critical to offset potential negative impacts of SARS-CoV-2 or future pandemic-related restrictions on HIV epidemic dynamics.

**Funding:**

Funding sources include Genome Canada and Genome BC, the Public Health Agency of Canada, the BC Centre for Excellence in HIV/AIDS, and the Canadian Institutes of Health Research Coronavirus Rapid Response Programme. Student funding includes a NSERC CREATE scholarship and a Canadian Institutes of Health Research graduate fellowship.

**Research in context**

**Evidence before this study**

We searched PubMed for articles relevant to COVID-19-related impacts on HIV-transmitting populations, published in any language, before March 18^th^, 2022. Several reports demonstrate coinciding changes in risk-related behaviours and rate of diagnosis for key HIV-transmitting populations in relation to changing attitudes and restrictions regarding COVID-19. COVID-19-related reductions in the availability of health services needed for HIV detection and management were commonly reported. At the same time, some populations experienced decreased opportunity to participate in risky activities, while others experienced pressures driving increased risky behaviour. Comprehensive phylogenetic investigation of the impacts of the reduction in health service availability on HIV transmission dynamics and detailed analysis of differing impacts between populations characterized by different risk factors been conducted have yet to be published.

**Added value of this study**

We conduct several phylogenetic analyses on HIV sequence data collected in British Columbia, Canada in order to quantify the impact of COVID-19-related health service shutdowns on HIV transmission dynamics across key populations characterized by differing risk factors. Our results reveal increased transmission following the implementation of COVID-19 restrictions in a limited number of transmission clusters associated with people who inject drugs.

**Implications of all the available evidence**

The downstream transmission impacts of gaps in health service ability can be substantial. Differing pressures, behaviours and needs between key populations should be carefully considered in restriction design and amendment. Increased vigilance and innovative targeted solutions are critical to offset potential negative impacts on HIV treatment and prevention stemming from not only COVID-19 restrictions, but also restrictions related to future pandemics or other major public health events.

Alt-text: Unlabelled box

## Introduction

As the transmission of SARS-CoV-2 became a global public health crisis in early 2020, the initial defense strategy chosen by many regions was to recommend and implement restrictions designed to drastically reduce levels of connectivity within and between communities. In order to interrupt transmission chains quickly and curtail exponential spread, restrictions were often constructed with broad application in mind and could have unintended consequences.^1^ Broad restrictions have been shown to disproportionately affect vulnerable subpopulations who rely on social, economic, and medical supports, the disruption of which can further exacerbate challenges such as food insecurity,^2^^,^^3^ gender-based violence,^4^^,^^5^ management of medical conditions,^6^^,^^7^ and drug use.^8^^,^^9^ The population of individuals living with or at risk of HIV infection is also vulnerable to such impacts, as the successful management and prevention of HIV often require access to clinics and services that may be shut down or limited in capacity as a result of COVID-19 restrictions. Without access, individuals can be left without diagnostic testing, viral load testing, antiretroviral therapy (ART), pre-exposure prophylaxis (PrEP), safe injection materials, condoms, and other resources crucial to keeping pathogen transmission controlled within at-risk communities. Reduced engagement with and availability of HIV treatment and prevention services in association with COVID-19 and its related restrictions has been documented in many countries,^9^, ^10^, ^11^, ^12^, ^13^, ^14^, ^15^, ^16^ in some cases coinciding with worsening HIV outcomes such as viral load rebound^15^ or progression to AIDS.^14^ Even in locations such as Australia with very few early interruptions to HIV care,^17^ both HIV tests^18^ and PrEP use^19^ reportedly decreased, perhaps due to reduced risk behaviours or hesitancy to risk COVID-19 infection by visiting a hospital or clinic. The effects of the COVID-19 pandemic on HIV care and epidemic control present a complicated challenge to global public health systems, and quantification of the downstream effects of this disruption is necessary for the design of effective countervailing strategies.

**Figure 1:**
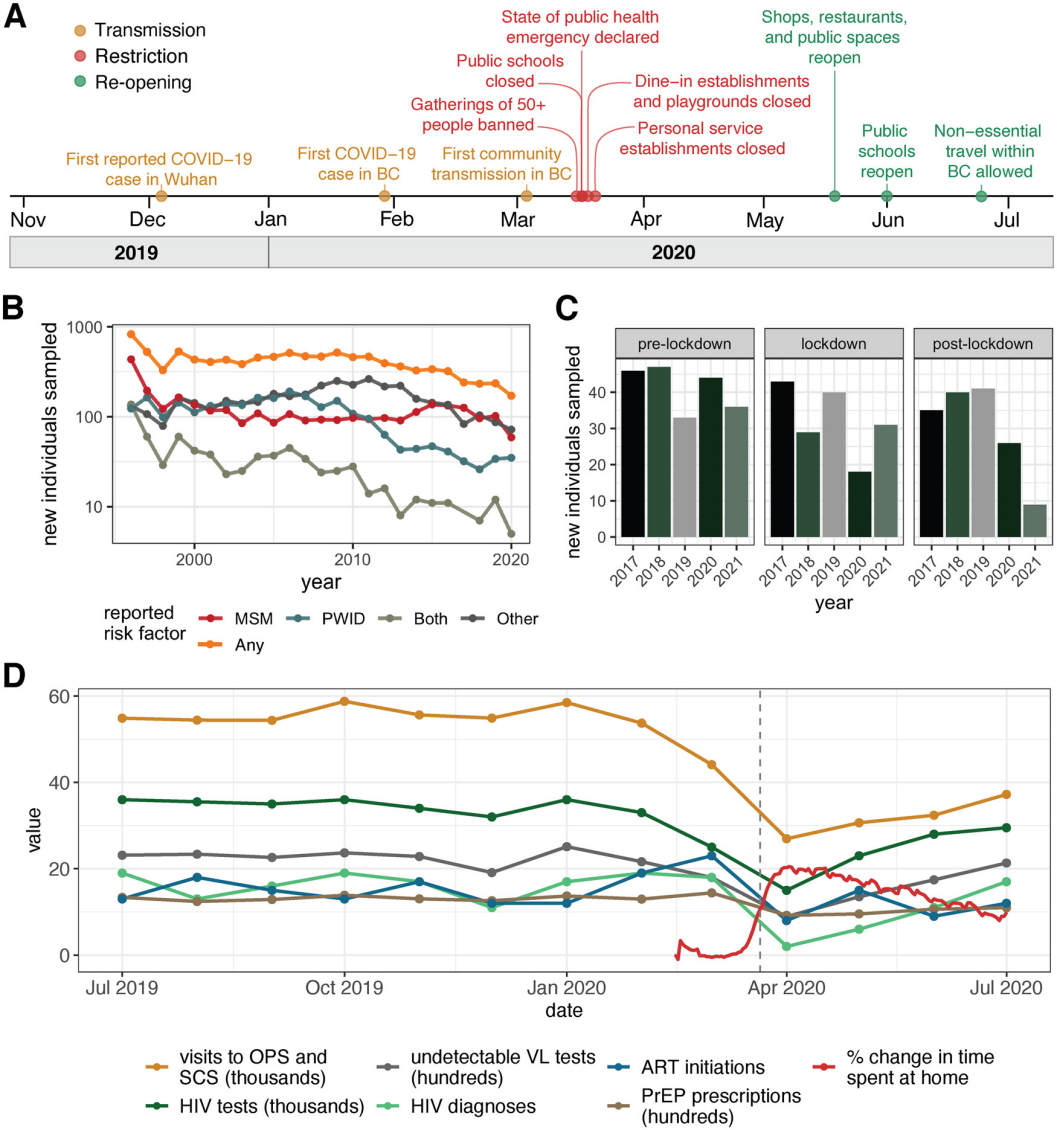
**COVID-19 related events and changes in HIV service engagement.** (A) Timeline of COVID-19-related events in British Columbia. The majority of restrictions were put in place during mid-March, 2020, culminating on March 21^st^. (B) Line plot showing the number of new individuals added to the dataset by year. Each line shows the number of individuals reporting a given risk factor, with the orange line delineating the total number of individuals per year, regardless of risk factor. “Both” indicates individuals who reported both MSM and PWID as possible routes of transmission. “Other” indicates either individuals with no reported risk factor information available or individuals who reported risk factors other than MSM or PWID, such as heterosexual sex, receipt of contaminated blood products or mother-to-child transmission. C) Bar plot showing the number of new individuals sequenced in each time period in each year. Pre-lockdown, lockdown, and post-lockdown represent the same 60-day time periods in each of the years, where pre-lockdown includes January 22–March 21, lockdown includes March 22–May 20 and post-lockdown includes May 21–July 19. It is important to note that since our dataset ends on June 4th 2021, the count for the post-lockdown period for 2021 is cut off here. D) Reduction in engagement with HIV prevention and management services following the implementation of lockdown restrictions coincides with increases in the amount of change to time spent in residential locations. The grey dashed line marks March 21^st^. Abbreviated markers of engagement include viral load (VL) tests yielding undetectable viral load, pre-exposure prophylaxis (PrEP) prescriptions, antiretroviral therapy (ART) initiations, and visits to overdose prevention services (OPS) and safe consumption sites (SCS). Change in time spent at home was assessed relative to baseline, as defined in the Google COVID-19 Mobility Reports data as the median value for a given day of the week from the five-week period between January 3, 2020 and February 6, 2020. The red line shown represents the 7-day rolling average of change in time spent at home. Data describing monthly engagement with HIV prevention and management services were collected by the BC-CfE Drug Treatment Program and the BC Centre for Disease Control (BCCDC). Data describing visits to OPS and SCS originated from the BCCDC Overdose Response Indicator Report. (For interpretation of the references to color in this figure legend, the reader is referred to the web version of this article.)

In the province of British Columbia (BC), Canada, several different types of restrictions amounting to the region's first “lockdown” were announced in March 2020,^20^ culminating on March 21^st^ (Figure 1A). On March 16^th^, health officials banned gatherings of more than 50 people and ordered bars and nightclubs to close. On March 17^th^, public schools were ordered to close and the province declared a public health emergency. March 20^th^ saw the closure of dine-in establishments and playgrounds, and personal service establishments were shut on March 21^st^. Although no official work-from-home order was put in place, many employers shifted their employees out of the office during mid-March, further contributing to reduced contact rates. During this time, many sites offering health-related services reduced their capacity and hours of operation. Even essential services that were encouraged to stay open, such as safe injection sites, underwent notable declines in availability as the necessary adaptations to reduce the chance of SARS-CoV-2 transmission were put in place. After approximately two months of lockdown, the reopening of shops, restaurants and public spaces was announced on May 19^th^, and public schools reopened on June 1^st^. By June 24^th^, BC was entering the third phase of its reopening plan, marking the beginning of an approximately three-month period with minimal restrictions before the “second wave” of transmission began in October 2020.

A potential positive side effect of the restrictions enacted in BC being followed by a dramatic increase in the amount of time spent at home is that for some populations at risk of HIV infection, contact rates were likely reduced, thus reducing the chance of transmission. This phenomenon has been documented in MSM in Australia^21^ and the UK,^22^ where reductions in the number of sexual partners were reported in association with COVID-19 awareness and restrictions. In BC, a study of sexual health service clients found that 31% reported a reduction in partners during the pandemic.^23^ However, populations characterized by non-sexual transmission routes such as shared needles may have experienced increased transmission risk as facilities such as safe injection sites reduced capacities whilst drug use,^9^^,^^24^ overdose calls,^25^^,^^26^ and risky transactional sex^27^ increased, possibly due to pandemic-related stressors^28^^,^^29^ and increased financial instability.^27^ Pandemic-related disruptions to health services have also been shown to contribute to increases in hepatitis C virus (HCV) transmission^30^ which is primarily transmitted by PWID.^31^ Additionally, willingness to seek sexual health services may have decreased, as described in a BC study where 66% of participants reported avoiding or delaying interaction with sexual health services during the pandemic.^23^ Consequently, we hypothesize that following the initial implementation of lockdown restrictions and reduced access to HIV management and prevention services in BC, key populations of individuals at increased risk for HIV acquisition will demonstrate differential trends in rates of transmission, some of which may not be outweighed by the effects of reduced contact rates. We, therefore, undertook a series of phylogenetic analyses to quantitatively approximate transmission preceding, during and following the implementations of restrictions.

## Methods

### Supporting data & statistical analyses

Data describing monthly engagement with HIV prevention and management services were collected by the BC-CfE Drug Treatment Program and the BC Centre for Disease Control (BCCDC). Data describing visits to Overdose Prevention Services (OPS) and Safe Consumption Sites (SCS) originated from the BCCDC Overdose Response Indicator Report.^32^ Movement trends were assessed using the Google COVID-19 Mobility Reports data.^33^ Change in time spent at home was assessed relative to baseline, as defined in the Google COVID-19 Mobility Reports data as the median value for a given day of the week from the five weeks between January 3, 2020, and February 6, 2020. Pandemic-related events used in the timeline seen in Figure 1A were selected from a similar timeline published by CTV News.^20^ Mann-Whitney tests quantified changes in service engagement from monthly data spanning August 2019 to March 2020 versus April 2020 to July 2020. The relationships between cluster growth or change in diversification rate and the proportion of risk factors reported within a cluster were quantified via Spearman correlation. All statistical analyses were performed in version 3.6.1 of the R statistical framework.^34^ R packages used include ape, cowplot, ggtree, lubridate, phangorn, phylobase, phytools, tidyverse, reshape2, and scales.

### Sequence dataset

38,408 partial *pol* HIV-1 sequences and associated metadata were collected from 10,386 individuals during drug-resistance genotyping tests performed by the BC Centre for Excellence in HIV/AIDS (BC-CfE) as part of routine clinical care following diagnosis between May 30^th^, 1996 and June 4^th^, 2021. The study population includes all individuals for which both at least one sequence and its collection date were available (*n* = 10,386), and the dataset is comprised of the earliest sequence available for each of these individuals. Recent estimates of the size of the total infected population in BC suggest that 84% of infected individuals are diagnosed and 85% of those individuals are on ART,^35^ indicating that somewhere between 71% and 85% of the total infected population in BC is sampled by our dataset. The number of new individuals added to the dataset declined over time, in concordance with enhanced epidemic control measures introduced throughout the 2000s^35^ (Figure 1B). In recent years, new individuals reporting only MSM risk outnumbered new individuals reporting only PWID risk (Figure 1B). None of the individuals sampled in our dataset was infected with more than one subtype.

The University of British Columbia – Providence Health Care Research Ethics Board granted ethical approval for this study (H17-01812).

### Phylogenetic analyses

The full sequence dataset was aligned with MAFFT^36^ and filtered to contain only the earliest sequence per individual, in order to focus phylogenetic inference on between-host evolution and avoid within-host inflation of lineage-level diversification rates. Surveillance drug resistance mutations were masked before maximum likelihood inference of 100 bootstrap phylogenies in IQ-TREE^37^ under a GTR+F+R10 model, as determined with ModelFinder.^38^ Transmission clusters were determined using a minimum size threshold of five, a 90% bootstrap threshold, and a phylogenetic distance threshold of 0.02 substitutions/site, as defined by Poon et al. .^39^ Cluster growth, branching events and lineage-level diversification rates were assessed in three 60-day time periods, including “pre-lockdown” (January 22–March 21, 2020), “lockdown” (March 22–May 20, 2020), and “post-lockdown” (May 21–July 19, 2020). In each time period, the three phylogenetic measures of transmission were evaluated across clusters and compared between groups of clusters associated with different risk factors. Each cluster was assigned a risk composition score based on the breakdown of risk factors reported by cluster members. The total number of sequences collected from newly sampled individuals during each of the time periods under study is shown in Figure 1C.

### Risk factor classification

Cluster risk composition was calculated as the proportion of PWID minus the proportion of MSM individuals in a cluster, such that a risk composition of 1 indicates 100% PWID reporting with no reporting of MSM and a risk composition of −1 suggests the opposite. Members of the same cluster may report different risk factors, and a single individual may report multiple risk factors, meaning that some clusters contain substantial risk factor overlap. Men who reported both sex with men and injection drug use were included both in the calculation of proportion PWID and in the calculation of proportion MSM. Stratifying clusters by risk composition allows analysis of trends across the distribution of risk-factor homogeneity and facilitates exclusion of ambiguous clusters when necessary. The proportions of individuals reporting different risk factors in a cluster were calculated using data collected after 2016, in order to appropriately represent current trends in transmission for each cluster. In analyses that sum cluster outcomes, binary classification of cluster risk factors was done by labelling all clusters with a cluster risk composition of −0.5 or less as MSM and all clusters with a cluster risk composition of 0.5 or more as PWID. Ambiguous clusters in between these thresholds were not included.

### Adjusted cluster growth

Cluster growth was calculated as the number of newly sampled cases to join a cluster during a given time period. For example, in *Supplementary Figure* S1A we would calculate the cluster growth to be 1 in time period A and 3 in time period B. Although biased by sampling proportion and recency of presentation to care, cluster growth is still valuable in estimating the level of transmission associated with different groups of individuals.^40^ Adjusted cluster growth was calculated as the number of new cases joining a cluster, normalized by cluster size and total newly sequenced individuals sampled (regardless of risk factor) during a given time period. Total adjusted cluster growth was determined by summing the adjusted cluster growth of clusters classified as MSM or PWID. Because we define cluster membership only once by summarizing phylogenetic links between individuals across all bootstraps, we do not evaluate variation in the observed adjusted growth of clusters.

### Adjusted number of branching events

Branching events, otherwise referred to as coalescent events, are bifurcations of a phylogenetic tree that suggest transmission as one lineage becomes two. Time-scaled phylogenetic trees can be used to estimate the timing of branching events, thus providing some indication of when transmissions are likely to have occurred based on the sequences and collection dates used to infer the phylogeny. For example, in *Supplementary Figure* S1B, there are three branching events: one dating to early March, one dating to mid-April and one dating to early May. These estimates are valuable in a public health context because sequences may be collected at any time after transmission, so by incorporating sequence data, it is possible to recover a more informed estimate of the true time of transmission than by using collection dates alone.

The median number of branching events associated with each cluster in each time period was determined from the ten most likely of 100 bootstraps inferred from alignments excluding all sequences predating 2017, done to increase the precision of time-scaling in LSD2.^41^ Rooting in LSD2 was done on all branches using constraints and single variance on branch lengths. To define which branching events were most likely to be associated with a certain risk factor, branching events were linked to clusters based on the cluster membership of the descendants of the branching node (*Suppl. Figure* S1B). Via this method, each branching node is also assigned an associated cluster risk composition score. Because clusters are often not entirely homogenous in terms of reported risk factors, this means some branching events will have some descendants reporting risk factors that differ from the overall risk factor classification of the event. For example, a node with descendants who are part of a predominantly MSM cluster will be linked to this cluster and its risk composition, even though it may lead to some PWID descendants (*Suppl. Figure* S1B, cluster 2). There were no cases of descendants being split between multiple clusters, although some descendant groups contained non-clustered sequences in addition to clustered sequences.

Adjusted branching events were calculated as the median number of branching events associated with a cluster across bootstraps within a given time period, normalized by size of the cluster and number of newly sequenced individuals sampled (regardless of risk factor) in a given time period. Confidence intervals on adjusted cluster medians were calculated across bootstraps. The adjusted sum of median branching events was calculated by totalling the adjusted median number of branching events inferred to be associated with clusters of each risk factor group during a given time period.

### Adjusted change in lineage-level diversification rate

Lineage-level diversification rate is a phylogenetically-derived and tip-weighted measure that describes lineages’ historical rate branching rate in a phylogeny, with emphasis on branching occurring closer to the present.^42^^,^^43^ Because each HIV branching event can be considered to be representative of the formation of a new lineage and thus a transmission event, lineage-level diversification rate serves as a proxy for transmission rate. For example, as illustrated in light green in *Suppl. Figure* S1C, tips that share recent common ancestry with many other tips will have higher diversification rates, suggesting rapid transmission. Conversely, as highlighted with dark green in *Suppl. Figure* S1C, tips preceded by longer branches with fewer descendants will have lower diversification rates, suggesting slower transmission, or possibly poorer sampling. This method of approximating transmission has previously been shown to be predictive of new HIV cases^44^ and concordant in the identification of clustering risk factors^45^ and is thus useful in the detection of populations at high risk for transmission.

In order to approximate the transmission rate, lineage-level diversification rates were calculated as in Jetz et al.^42^ for each phylogenetic tip in the ten most likely bootstraps. The median change in diversification rate by cluster was determined by taking the mean tip-level change between time periods in each cluster in each bootstrap, then calculating the median of these values for each cluster across bootstraps. The mean was used rather than the median in the initial summarization in order to increase detection of change since the majority of tips experienced zero change due to the relatively short 60-day observation periods and reduced sampling proportion. Adjusted change in diversification rate was calculated by normalizing the median values for each cluster by cluster size and the number of new sequences collected within a given time period (regardless of risk factor). Confidence intervals on adjusted cluster medians were calculated across bootstraps. The total adjusted median change in diversification rate was determined by summing the adjusted median change in diversification rate associated with clusters classified as being associated with each risk factor. Change in lineage-level diversification rates was used to approximate increases in transmission rate rather than raw diversification rate because diversification rate is expected to increase over time as the likelihood of new sequences being in close phylogenetic proximity to existing sequences rises with increased sampling. Thus, it is more appropriate to compare the magnitude of increase rather than the raw value when making comparisons across time. Variation in lineage-level diversification rate across all tips included in the full dataset can be seen in *Suppl. Figure* S2.

### Role of the funding source

The BC Centre for Excellence in HIV/AIDS was involved in data collection. All other funding sources had no direct role in study design, data collection, data analysis, interpretation, writing of the manuscript or decision to submit for publication.

## Results

### Reduction in HIV service engagement following lockdown

Individual movement data revealed that following the lockdown restriction announcements culminating on March 21^st^, time spent at home increased markedly, likely leading to reduced contact rates (Figure 1D). Also in April, there were statistically significant reductions in markers of engagement with HIV services, including ART initiation (Mann-Whitney *p* = 0.030), PrEP prescription dispensations (Mann-Whitney *p* = 0.00026), undetectable plasma viral load tests (Mann-Whitney *p* = 0.0037), HIV tests (Mann-Whitney *p* = 0.0057), new HIV diagnoses (Mann-Whitney *p* = 0.049), and visits to Overdose Prevention Services (OPS) and Safe Consumption Sites (SCS) (Mann-Whitney *p* = 0.00017) (Figure 1D). Importantly, as the lockdown period began, the BC-CfE recommended a reduction in viral load testing frequency in patients with long-term viral load suppression, to preserve SARS-CoV-2 testing capacity. This directive is likely responsible for at least some of the reduction in undetectable viral load tests. Post-lockdown, all markers of engagement rebounded to below pre-lockdown levels. New HIV diagnoses in the province remained on an overall declining course.

### Transmission clusters

**Figure 2:**
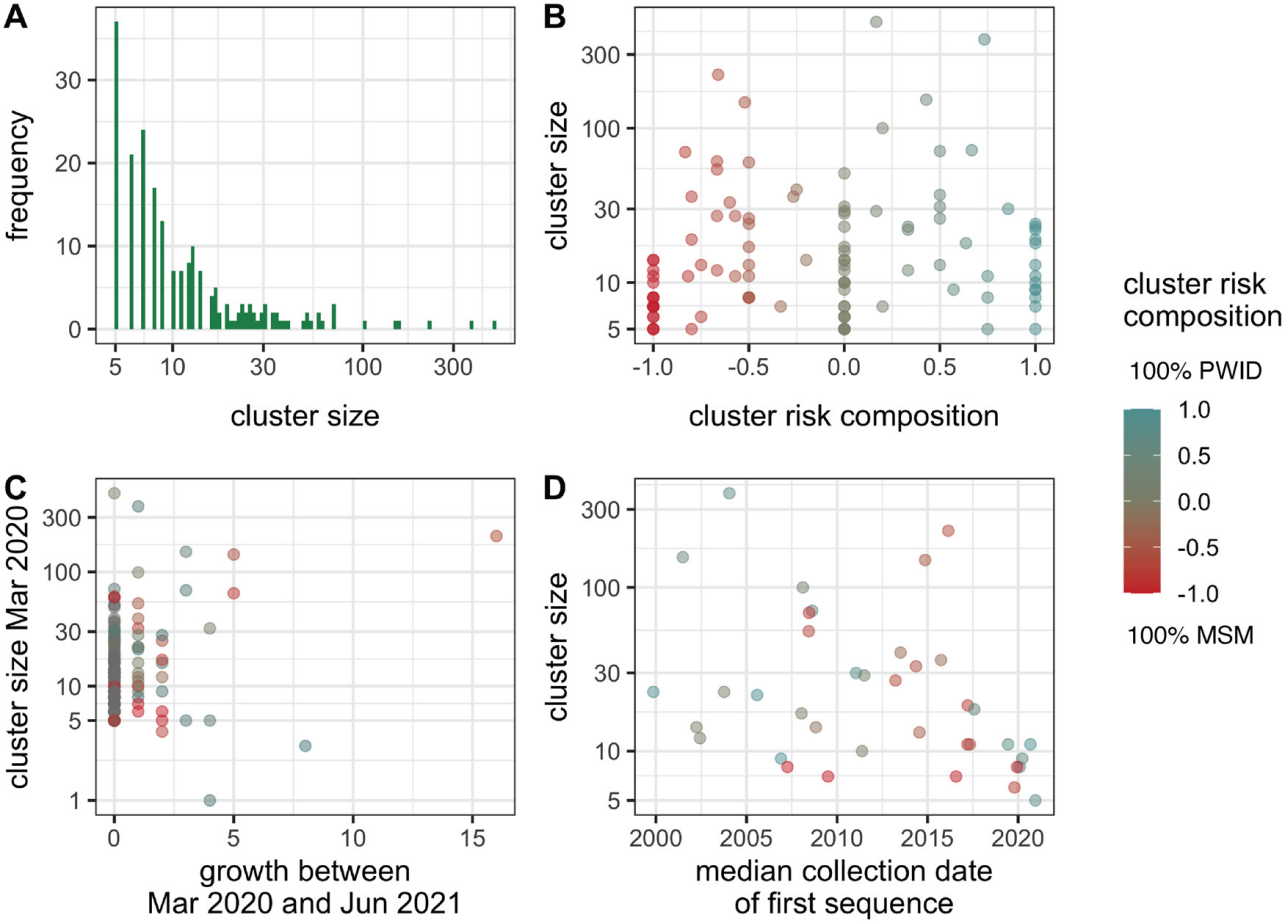
**Transmission cluster characteristics**. A) Distribution of cluster sizes for all inferred clusters (*n* = 208). B) Distribution of cluster risk composition across different cluster sizes, shown for all clusters for which recent risk factor information was available for calculation of cluster risk composition (those that grew after 2016, *n* = 105). C) Comparison of cluster growth between the implementation of lockdown restrictions (March 21^st^, 2020) and the end of the collection period for the dataset (June 4^th,^ 2021), versus cluster size before restriction implementation. Observations are coloured by cluster risk factor composition and thus only include the clusters for which this information is available (*n* = 105). D) Median collection date of the first sequence collected for individuals within a cluster, shown for all clusters that demonstrated growth in panel C (*n* = 38). Observations are coloured by cluster risk factor composition. (For interpretation of the references to color in this figure legend, the reader is referred to the web version of this article.)

208 clusters were inferred, containing 4311 individuals in total. Clusters varied in size, ranging from five to 488 individuals (median size = 9, Figure 2A). 105 clusters showed growth since 2016, making the calculation of recent cluster risk composition possible for this subset. 68 clusters (26 PWID, 42 MSM) could be binarily classified as primarily PWID or primarily MSM based on the −0.5 and 0.5 thresholds described above. Clusters also varied in risk composition (Figure 2B), with no significant difference in size between risk groups after binary classification (Mann-Whitney *p* = 0.26). A subset of the total number of clusters ranging in risk factor composition demonstrated growth following the implementation of lockdown restrictions (*n* = 38 [13 PWID, 15 MSM], Figure 2C) and thus form the focus of downstream analyses. The median collection date of the first sequence associated with cluster members was generally diverse across cluster sizes and cluster risk composition, although clusters with the earliest median dates (pre-2005) were primarily shifted towards higher PWID composition (Figure 2D). There was a moderate and significant negative relationship between the first sequence date and cluster size (Spearman r = −0.43, *p* = 0.0066), but many clusters of varying sizes deviate from this relationship (*Suppl. Figure* S3).

### Transmission changes following lockdown

New diagnoses decreased from 44 during the pre-lockdown period to 18 during lockdown and began to rebound thereafter (26 post-lockdown). As suggested by the dip in service engagement during the lockdown, it is likely that the sampling proportion of newly diagnosed infections also declined during this time, meaning that the decrease in diagnoses does not necessarily indicate a proportional decrease in transmission. Further phylogenetic analyses support this idea and suggest that the magnitude of transmission occurring differs between risk groups.

**Figure 3:**
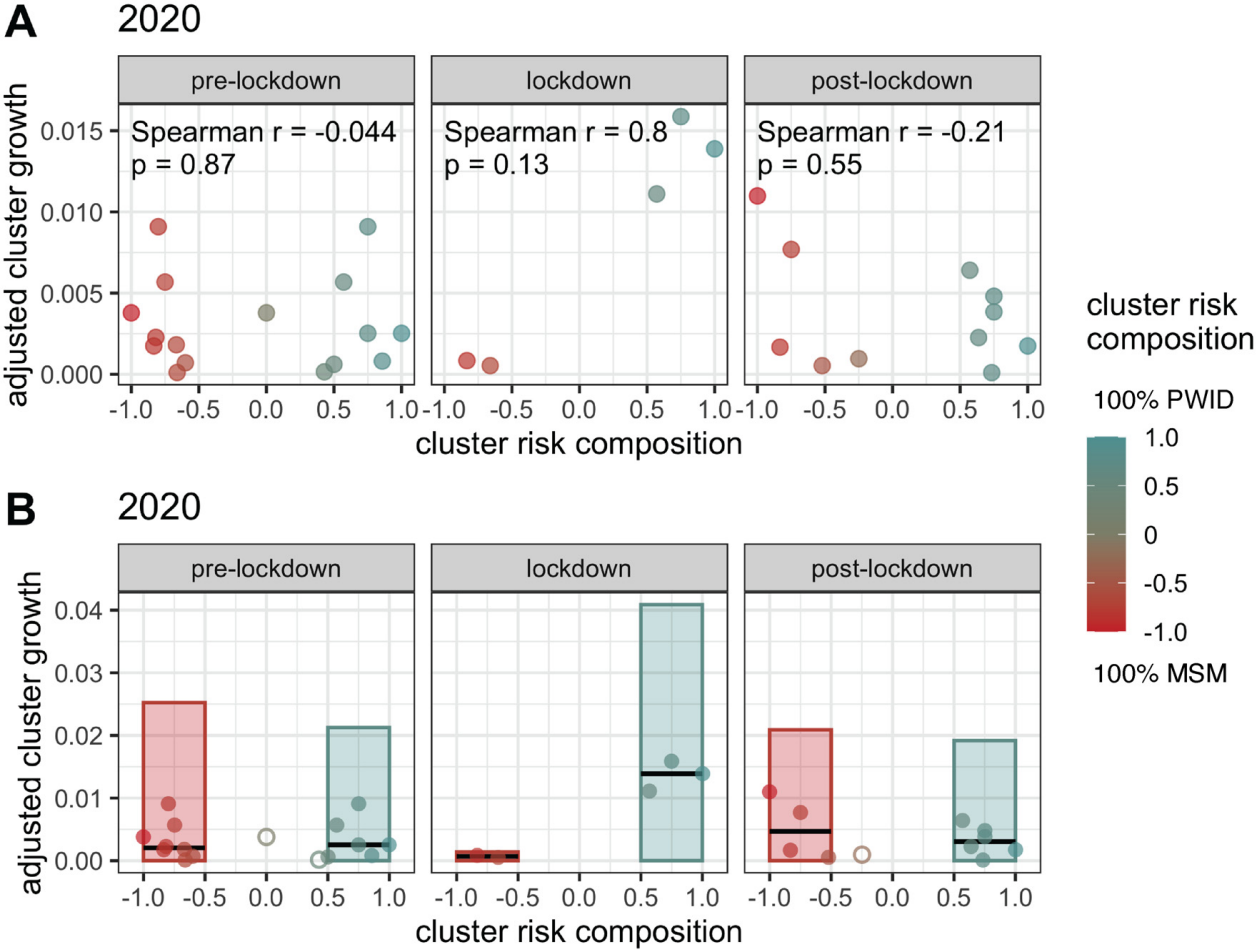
**Differences in adjusted cluster growth by risk factor composition.** A) Adjusted cluster growth versus cluster risk composition, defined as the proportion of PWID cluster members minus the proportion of MSM cluster members. Observations are coloured by cluster risk composition, such that the reddest clusters have the highest proportion of MSM individuals and the bluest clusters have the highest proportion PWID individuals. Adjusted cluster growth refers to the amount of cluster growth, normalized by cluster size and number of newly sequenced individuals sampled during a given time period. Only clusters that experienced growth during a given time period are shown. B) Bars represent total adjusted cluster growth seen in all clusters associated with each risk factor in a given time period. In this analysis, binary classification of cluster risk factors was done by labelling clusters with a risk composition of −0.5 or less as MSM and clusters with a risk composition of 0.5 or more as PWID. Ambiguous clusters in between these thresholds, marked by open circles, were not included in the group totals. Closed circles represent clusters contributing to the risk group totals. Black horizontal lines represent the median adjusted cluster growth of the closed circles associated with each risk group in each time period. (For interpretation of the references to color in this figure legend, the reader is referred to the web version of this article.)

When normalized by cluster size and total number of new diagnoses in each month, cluster growth demonstrated a peak in the strength of its relationship with cluster risk factor composition during the lockdown period that was more than 3.2 standard deviations greater than the mean effect size seen across all other time periods (Figure 3A). Although none of the results were significant, the pre-lockdown and post-lockdown periods in 2020 show negative correlations between adjusted cluster growth and cluster risk composition (Spearman r = −0.044, −0.21; *p* = 0.87, 0.55), while a strong positive effect size exists during the lockdown period (Spearman r = 0.80, *p* = 0.13), indicating higher growth as cluster risk composition shifts towards 100% PWID. Furthermore, a strong positive correlation is not seen in any of the three equivalent time periods during the previous three years (*Suppl. Figure* S4). Analyses of both total and median adjusted cluster growth reveal that MSM-dominant clusters experience a notable decline to near zero during the lockdown, while PWID populations peak at a level unseen during any of the equivalent time periods in the previous three years (Figure 3B, *Suppl. Figure* S5).

**Figure 4:**
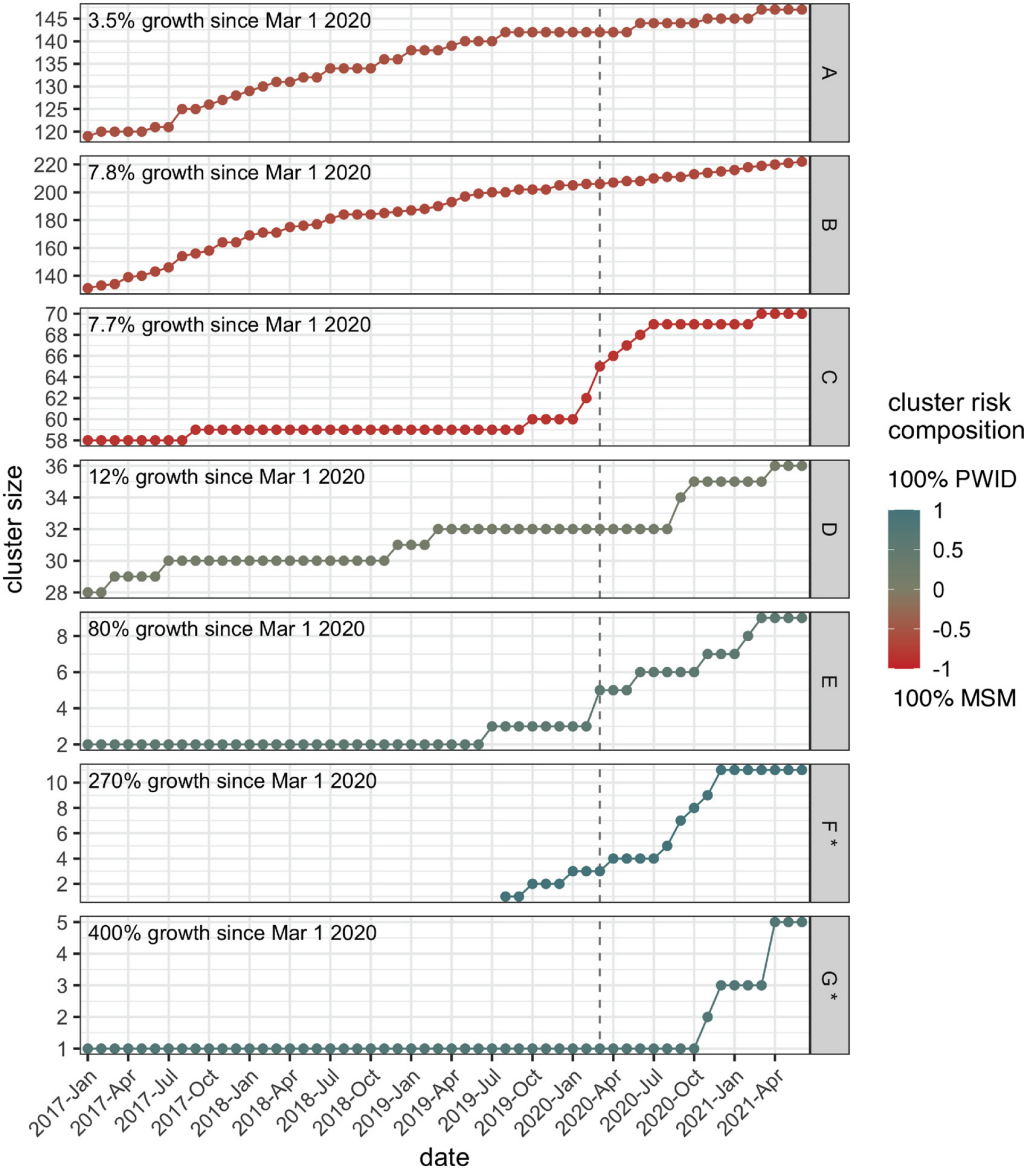
**Long-term cluster growth for all clusters that grew by four cases or more following lockdown in March 2020 until June 2021.** The plots are shown in ascending order, based on the percent increase in cluster size seen between March 1^st^, 2020 and June 4^th^, 2021. Unlike the previous figures, raw unadjusted cluster growth is shown. Colour indicates cluster risk composition of reported risk factors within a cluster, specifically the proportion of MSM subtracted from the proportion of PWID, such that the red-hued clusters have the highest proportion of MSM individuals and the blue-hued clusters have the highest proportion PWID individuals. Arbitrary cluster identification numbers are shown in the strip label. The clusters marked with an asterisk were initially identified after the implementation of lockdown restrictions. The grey dashed line marks March 2020, the month lockdown restrictions were put in place. (For interpretation of the references to color in this figure legend, the reader is referred to the web version of this article.)

Looking at individual clusters on a more long-term scale revealed similar trends – of all the clusters that grew by four or more cases between March 21^st^, 2020 and June 4^th^, 2021, those with higher PWID risk composition generally underwent greater percentage growth (Figure 4). These clusters were much smaller (size 5–10) than the MSM-dominant clusters (size 70–147), so it is not unexpected that any number of new cases would result in high percentage growth. However, since we found no overall difference in cluster size between the two groups of clusters (Mann-Whitney, *p* = 0.26), it is notable that of the top-growing clusters shown in Figure 4, all the MSM-dominant clusters were large and the PWID-dominant clusters were small. Since the likelihood of a cluster growing increases with its size, growth of four or more cases in a small cluster can be said to be more concerning than the same growth in a cluster composed of hundreds of individuals. Furthermore, the only new clusters identified during this time display PWID-rich risk composition (0.75, 1). Examining long-term trends also allowed observation of current growth in the context of past growth. The two large MSM-predominant clusters (A and B) shown in Figure 4 demonstrate a history of steady growth that continues at a relatively similar pace following the implementation of restrictions. Conversely, the three small PWID-dominant clusters (E-G), and the mid-size MSM-dominant cluster (C) demonstrate sharp increases in growth near the time of restriction implementation, suggesting that transmission dynamics related to these clusters are likely to have changed (Figure 4). However, clusters C and E display increases in growth rate that begin slightly before restriction implementation and then persist, thus complicating attribution of any changes purely to the implementation of restrictions. Similarly, Cluster D, a mid-size cluster of mixed risk composition, shows a history of sporadic growth that continues at slightly increased levels following lockdown, making it difficult to determine the extent to which this change may be related to the impact of restrictions (Figure 4). Notably, many of these clusters continued to experience elevated growth well beyond the initial period of instability in health service availability, indicating that the effects of such gaps can be long-term and difficult to counterbalance, thus expanding the potential for negative impact. Phylogenies for the top-growing clusters seen in Figure 4 can be found in *Suppl. Figure* S6. Cluster size across the observed time periods can be found in *Suppl. Table* S1.

**Figure 5:**
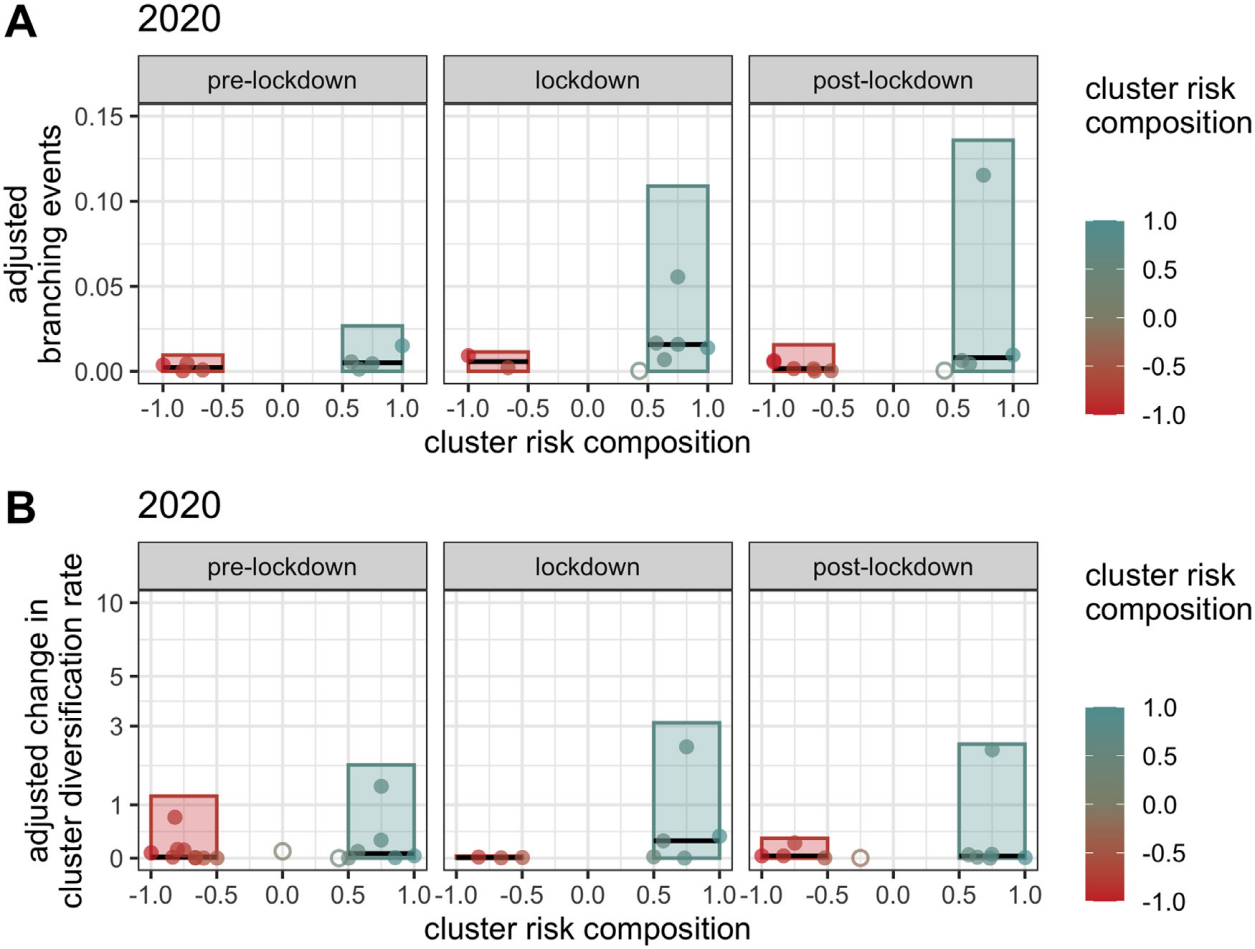
**Branching events and cluster change in diversification rate by risk factor composition.** Bars represent the total number of A) total daily median branching events inferred to be associated with clusters of each risk factor, normalized by cluster size and number of newly sequenced individuals during a given time period or B) total cluster median change in individual diversification rate between the beginning and end dates of a given time period, adjusted for cluster size and number of newly sequenced individuals in that time period. Observations are coloured by cluster risk composition, such that the reddest clusters have the highest proportion of MSM individuals and the bluest clusters have the highest proportion PWID individuals. Only non-zero values are shown. Binary classification of cluster risk factors was done by labelling clusters with a risk composition of −0.5 or less as MSM and those with a risk composition of 0.5 or more as PWID. Ambiguous clusters in between these thresholds, marked by open circles, were not included in the group totals. Closed circles represent clusters contributing to the risk group totals. Black horizontal lines represent the median of the closed circles associated with each risk group in each time period. In order to capture the branching events more likely to be associated with a certain risk factor, each event was assigned the risk factor composition of the cluster its descendants were members of, effectively linking each branching event to a cluster. (For interpretation of the references to color in this figure legend, the reader is referred to the web version of this article.)

The level of transmission occurring in association with MSM populations versus PWID populations was further explored via calculation of the number of cluster-associated branching events during each time period. When adjusted by cluster size and the total number of new diagnoses in each time period, the total number of putative transmissions associated with clusters showing higher PWID composition increased markedly during lockdown and continued to increase post-lockdown, although the median number of adjusted branching events decreased post-lockdown (Figure 5A). Analysis of individual clusters reveals the post-lockdown increase in branching events to be driven by a single rapidly growing cluster (Cluster G), while the remainder return to approximately pre-lockdown levels (Figure 5A). The levels of branching events seen during lockdown and post-lockdown were unmatched during the equivalent time periods in the previous three years (Figure 5A, *Suppl. Figure* S7). Clusters showing higher MSM composition were linked to similar numbers of branching events across the three time periods (Figure 5A). Variation in the adjusted number of branching events associated with clusters in each time period across bootstraps was generally minimal (*Suppl. Figure* S8).

Accelerated growth in clusters characterized by PWID versus MSM populations was further demonstrated by a comparison of the total median change in lineage-level diversification rates shown by cluster members following the announcement of lockdown restrictions, relative to cluster size and the total number of new diagnoses (Figure 5B). The total adjusted change in diversification rate in PWID-associated clusters increased during lockdown, reaching a level unseen in either group of clusters in the equivalent time periods over the preceding three years (Figure 5B, *Suppl. Figure* S9). Total change in diversification rate remained high in the post-lockdown period for PWID-associated clusters, again driven by a single cluster while the remainder return to pre-lockdown levels. This cluster is notably not the same cluster driving the high total value of branching events in the 2020 post-lockdown period, nor is it one of the clusters A-G demonstrating the most raw sampled growth in Figure 4. However, this cluster (Cluster AE) is the cluster showing the highest adjusted cluster growth during lockdown in 2020 (Figure 3). This result highlights the importance of using multiple phylogenetic approaches to monitor transmission, as some approaches may detect increased transmission in circumstances where others cannot. The total change in diversification rate for MSM-associated clusters did not differ drastically between time periods, but it did dip slightly during lockdown (Figure 5B). Variation in the change in diversification rates seen for tips in clusters in each time period across bootstraps was generally minimal (*Suppl. Figure* S10).

## Discussion

Our results support the theory that public health measures aiming to reduce transmission of SARS-CoV-2 early in the pandemic resulted in an increase in time spent at home, coinciding with a significant reduction in engagement in HIV prevention and management services. Our analyses further reveal increased transmission following the implementation of said public health measures in a limited number of PWID-associated transmission clusters. These clusters showed high growth rates, increased number of linked phylogenetic branching events, and elevated cluster median change in individual viral diversification rates during, and in some cases following, the lockdown period. Although these findings apply to a small number of clusters with relatively few members, the observed changes in transmission dynamics remain concerning. Small amounts of growth in larger clusters can occur even when the cluster is well-controlled overall, but the same amount of growth in a smaller cluster is more likely to indicate accelerated transmission or an increased chance of at least one individual contributing to multiple onward transmissions. Furthermore, due to the exponential nature of viral transmission, rapid and early response is crucial to epidemic control and prudent use of resources, making changes in small clusters worthy of attention. Evidence from the BC phylogenetic monitoring system extending beyond our dataset to the present demonstrates this; at least three small, PWID-dominated clusters that began accelerated growth near the implementation of restrictions have continued to grow rapidly well into 2022.

An important caveat of this study is that the dataset consists of one sequence for each new diagnosis, not for each new transmission. Fewer new infections may have been diagnosed and sequenced relative to pre-pandemic levels,^46^ meaning our approximations of transmission and cluster growth are likely to be underestimated. Additionally, differences or pandemic-related changes in contact tracing success may have had differential influence on key population sampling rates. Despite these factors, the level of transmission detected in several key population clusters remains concerning. A second caveat is that because sequences were collected as part of routine clinical care, the dates associated with the sequences reflect the date of first detectable viral load rather than the date of diagnosis or the date of infection, thus possibly introducing a varying degree of uncertainty to the timing of cluster growth, putative transmissions, and changes in diversification rate. Finally, although we aim to consider risk factor overlap via cluster risk composition, increases in overlapping risk behaviours linked to pandemic pressures (e.g., increased risky sex among PWID^27^) may not necessarily be captured by our dataset.

Differential transmission trends between MSM and PWID clusters here identified could be due to differences in the risk-behaviour patterns and medical supports required by individuals in these populations. While many MSM reduced sexual contact as a response to the pandemic and its related public health measures,^21^^,^^22^ PWID have shown increases in transmission-related behaviours.^9^^,^^24^, ^25^, ^26^ Additionally, anecdotal evidence from public health teams and healthcare providers in British Columbia suggests that PWID populations became more difficult to reach during the initial period of the pandemic; a combination of factors including increased housing instability and diminished trust increased obstacles to making contact and engaging effectively with individuals who would normally receive regular care preventive of transmission or infection. Our results are also consistent with concerning increases in growth seen in a small number of clusters following the implementation of pandemic restrictions, as noted by public health experts who monitor HIV transmission in BC. A modelling study preprint focused on the impacts of COVID-19 on the MSM population further supports the idea that opposing trends in new infections may result from different levels of risk behaviour.^47^ They found that a 25% reduction in sexual partners resulted in a 12.2% drop in new HIV infections over the following year, but in the absence of changes to sexual behaviour, the combined effect of disruptions to prevention-related services and behaviours was a 10.5% increase in new HIV infections.^47^ Similarly, a recent report from West Virginia, USA showed that suspension of a county syringe service program and COVID-19-related closures to other services needed by PWID were followed by notable local increases in HIV diagnoses.^48^ Another possible reason for transmission differences between key populations is that although all groups experienced reductions in the availability of the services they access, services such as safe injection sites are designed to be accessed with much greater frequency than other services such as clinics or testing sites, meaning the daily impact of closure or reduced capacity could accumulate much faster.

Evidence from other locations indicates that when restrictions are minimized, adjusted or adapted to, the reduction in access to services needed by PWID may be smaller. In Sweden, where the initial COVID-19 response was much less restrictive than in other countries, HIV tests did decrease, but measures of engagement with needle exchange programs remained stable or increased; 85.3% of participants reported sufficient access to safe injection equipment and only 10.3% reported less access to out-patient appointments.^49^ This suggests that although demand for support services during the initial months of the pandemic may have increased, the absence of a Swedish lockdown minimized interference with the medical needs of the population. Similarly, in Austria, regulations regarding opioid substitution therapy (OST) prescriptions were temporarily eased during lockdown in order to allow continued adherence, a choice thus far followed by no observed change in OST-related consumption patterns.^50^ However, minimizing or targeted loosening of restrictions may be unfeasible depending on the level of government cooperation and pandemic severity, making adaptations such as pre-packing supplies before distribution, providing delivery services or increasing the number of supplies given out at once^16^ a more viable option.

Although the long-term effects of disruptions to engagement with HIV care services are yet to be seen, mathematical modelling studies estimate the negative impacts to be substantial.^51^, ^52^, ^53^, ^54^ A 10% to 60% increase in HIV-related deaths could stem from disruptions to ART^51^^,^^52^ and viral suppression.^53^ Increases in HIV infection rates ranging from 5% to 15.7% have also been predicted in relation to reductions in health services^54^ and condom use.^53^ Due to the exponential nature of viral transmission, an increase of any magnitude in HIV infections can have downstream effects requiring much more resources to control than would be necessary for primary prevention. Thus, preserving engagement with HIV care is crucial to resource-efficient protection that limits the harm experienced by the at-risk community.

Maintaining engagement with HIV prevention and management services is crucial to preventing regression in epidemic control and unnecessary detriment to at-risk populations. In particular, differing pressures, behaviours and needs between key populations should be carefully considered prospectively, or at minimum as an amendment to restriction implementation. Increased vigilance and innovative targeted solutions are critical to offset potential negative impacts on HIV treatment and prevention stemming from not only COVID-19 restrictions but also restrictions related to future pandemics or other major public health events.

## Contributors

R.L.M., J.B.J. and J.S.G.M. are responsible for conceptualization. R.L.M. and J.B.J. devised the methodological approach, and were involved in direct access and verification of the data. R.L.M. conducted all analyses and visualization. Some analyses were conducted using code either written by or adapted from A.M. and J.B.J. The original manuscript draft was written by R.L.M. and reviewed and edited by V.M., J.T., S.S., J.H., R.H.L., J.W., R.B., and J.S.G.M., with significant editing contributions from A.M. and J.B.J.

## Data sharing statement

Due to the confidential nature of population level HIV sequence data sets and the criminalization of HIV transmission in Canada, the HIV sequences and associated meta data will not be made publicly available. All other data are publicly available.

## Declaration of interests

The authors have no conflicts of interest to declare.

## Appendix Supplementary materials

Image, application 1
