# Supplementary material for: Impact of SARS-CoV-2 lockdown on expansion of HIV transmission clusters among key populations: A retrospective phylogenetic analysis
Source: Lancet Reg Health Am. 2022 Sep 23;16:100369. doi: 10.1016/j.lana.2022.100369 (PMC9500205; doi:10.1016/j.lana.2022.100369)
Supplement: Supplementary file 1 [file mmc1.pdf]

Supplementary Appendix

Figure S1: Transmission measure examples....................2

Figure S2: Representative bootstrap tree colored by lineage-level diversification rate....................3

Figure S3: Distribution of sequence collection dates within clusters....................4

Figure S4: Differences in adjusted cluster growth by cluster risk factor composition in 2017 to 2019....................5

Figure S5: Differences in total adjusted cluster growth by risk factor in 2017 to 2019....................6

Figure S6: Phylogenetic trees for top-growing clusters....................7

Figure S7: Branching events in 2017 to 2019, by risk factor....................8

Figure S8: Variation in adjusted branching events by cluster across bootstraps....................9

Figure S9: Cluster change in diversification rates within each time period in 2017 to 2019, by risk factor....................10

Figure S10: Variation in change in lineage-level diversification rate by cluster across bootstraps....................11

Table S1: Cluster size during each 60-day time period in each year....................12

Supplementary Figures

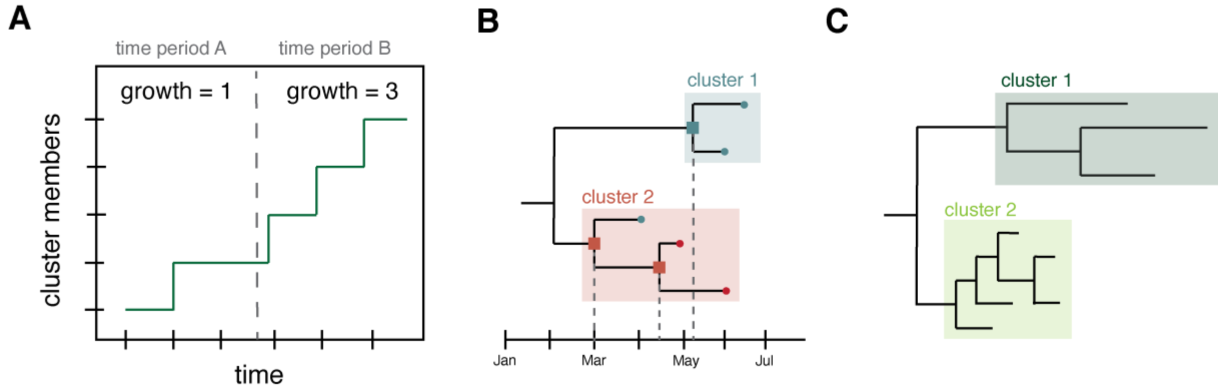

Figure S1. Transmission measure examples. A) Example of how cluster growth is calculated across different time periods. Here, cluster growth in time period A is one and cluster growth during time period B is three. B) Illustration of how branching events are linked to specific dates and clusters. By time-scaling the phylogeny, the times at which branching is inferred to occur can be observed. Here, there are three branching events: one dating to early March, one dating to mid-April and one dating to early May. We linked branching events to clusters based on the cluster membership of the tips descending from each branching node. Here, the most recent branching event leads to descendants in cluster 1, so the event and its timing will be linked to cluster 1 and its risk composition score. The earliest two branching events all result in descendants who are members of cluster 2, so both these branching events will both be linked to this cluster and its risk composition score, even though their descendants differ in risk composition individually. C) Example illustration of how differences in phylogenetic branching result in differing lineage-level diversification rates. As highlighted in light green, tips that share recent common ancestry with many other tips will have higher diversification rates, suggesting rapid transmission. Conversely, as highlighted with dark green, tips preceded by longer branches with fewer descendants will have lower diversification rates, suggesting slower transmission, or possibly poorer sampling. In this case we would expect cluster 1 to have a much lower median diversification rate across tips than cluster 2.

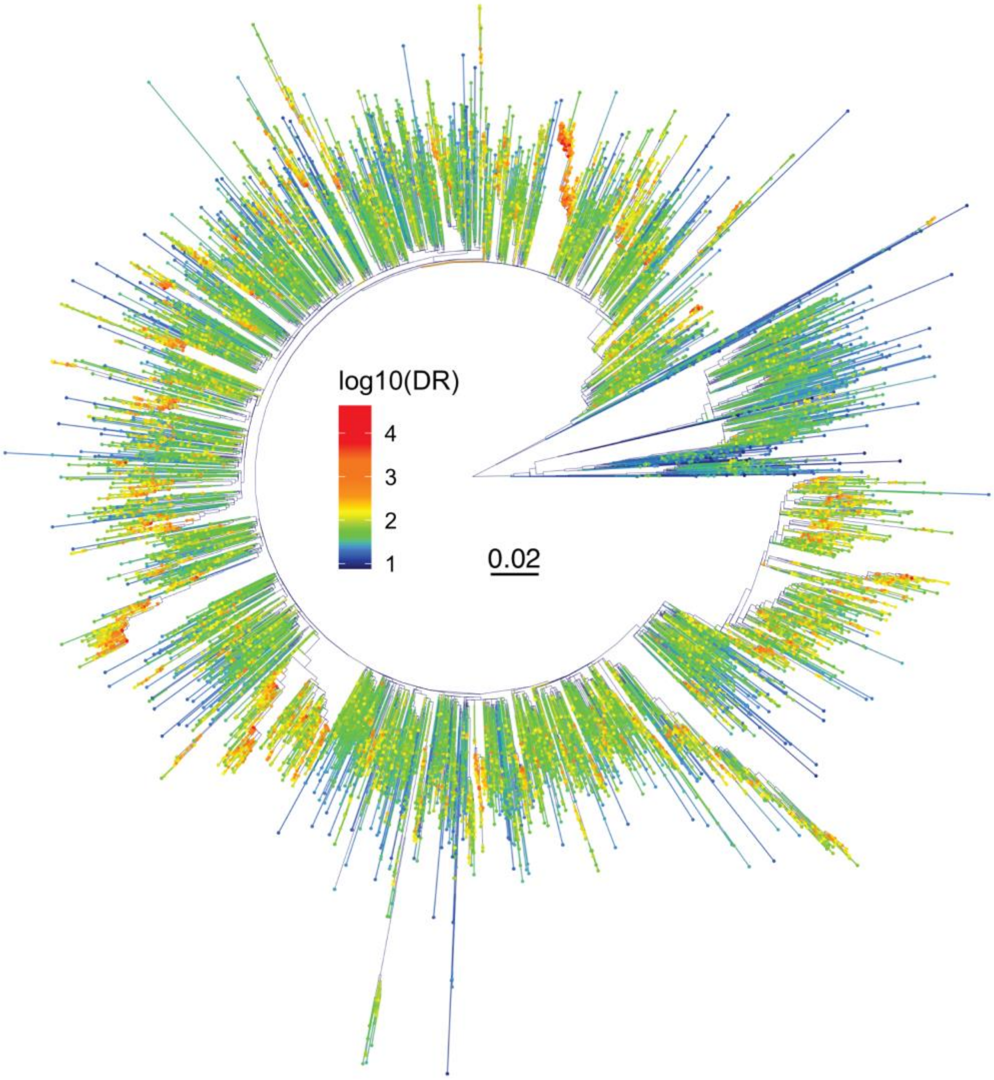

Figure S2. Representative tree coloured by lineage-level diversification rate. Highest likelihood tree of the 100 bootstraps, colored by log lineage-level diversification rate (DR). Warmer colors and thicker edges highlight high diversification rates, suggesting rapid transmission.

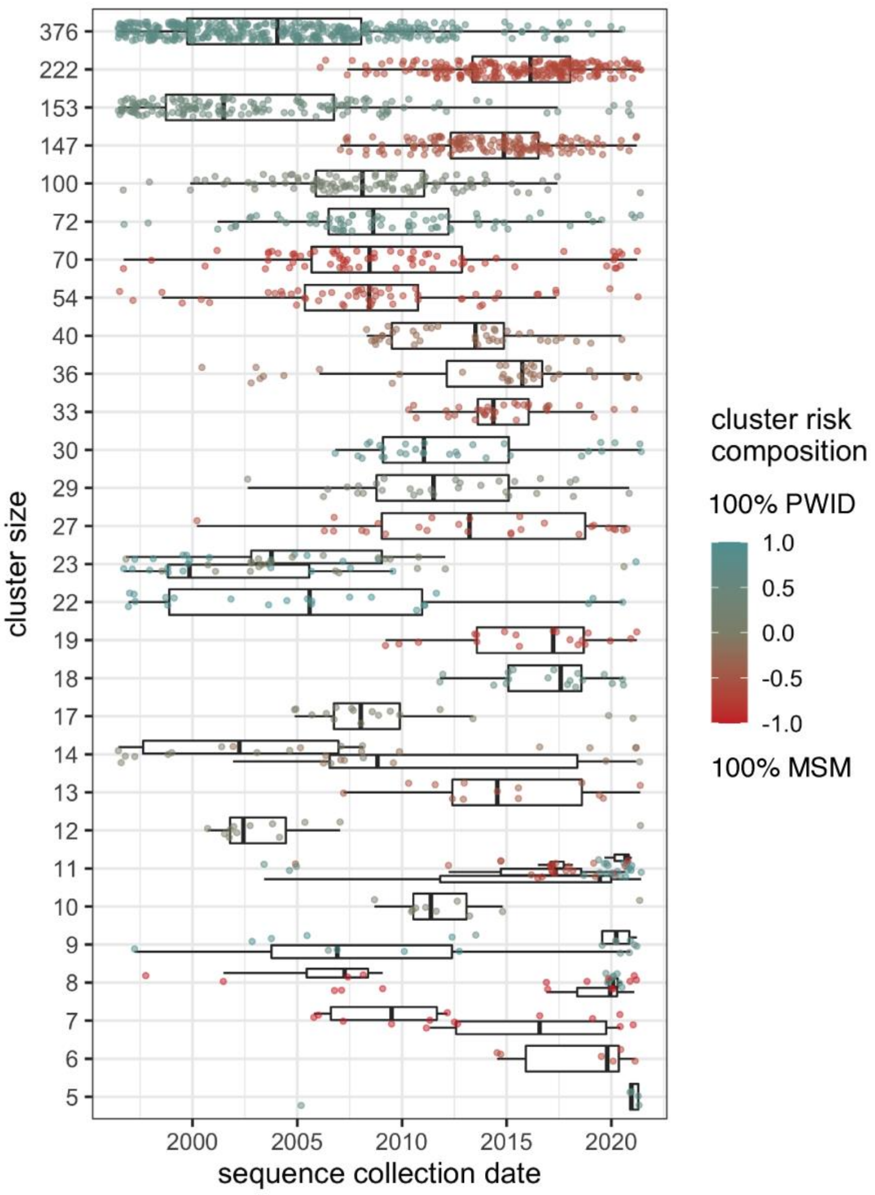

Figure S3. Distribution of sequence collection dates within clusters. Each box represents a cluster and each dot represents the date of collection of the first sequence associated with a cluster member. Dots are coloured by risk factor composition.

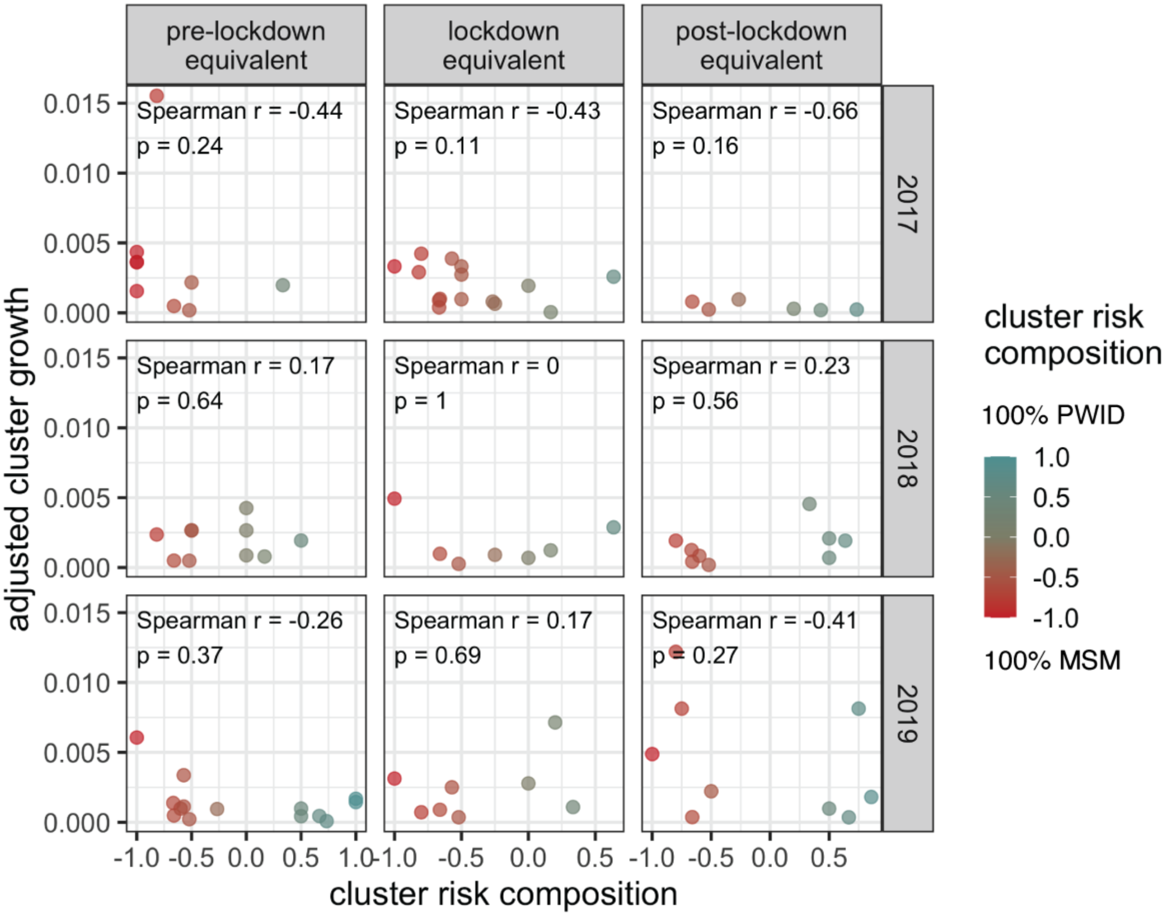

Figure S4. Differences in adjusted cluster growth by cluster risk factor composition in 2017 to 2019. Adjusted cluster growth versus cluster risk composition, defined as the proportion of PWID cluster members minus the proportion of MSM cluster members. Observations were selected from periods between the same dates as the time periods studied in 2020, creating three "equivalent" time periods. Observations are coloured by relative risk proportion, such that the red-hued clusters have the highest proportion of MSM and the blue-hued clusters have the highest proportion PWID. Adjusted cluster growth refers to the number of new cases in a cluster, normalized by cluster size and total new diagnoses during a given time period.

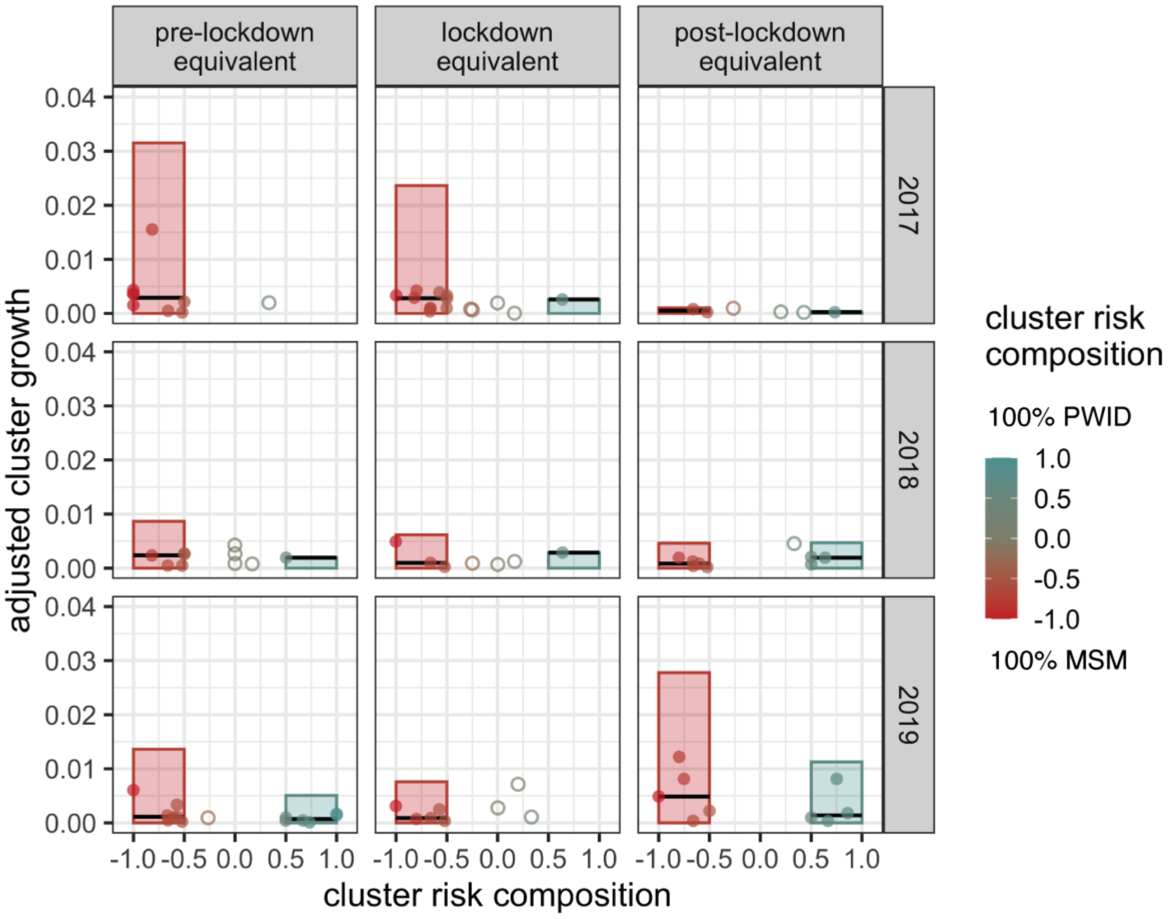

Figure S5. Differences in total adjusted cluster growth by risk factor composition in 2017 to 2019. Bars represent the total adjusted cluster growth seen in all clusters associated with each risk factor in a given time period. Observations were selected from periods between the same dates as the time periods studied in 2020, creating three "equivalent" time periods. Binary classification of cluster risk factors was done by labelling all clusters with a proportion of PWID minus proportion MSM of -0.5 or less as MSM and all clusters with a proportion of PWID minus proportion MSM of 0.5 or more as PWID. Ambiguous clusters in between these thresholds were not included in the group totals. Closed circles represent clusters contributing to the risk group totals. Black horizontal lines represent the median adjusted cluster growth of the closed circles associated with each risk group in each time period.

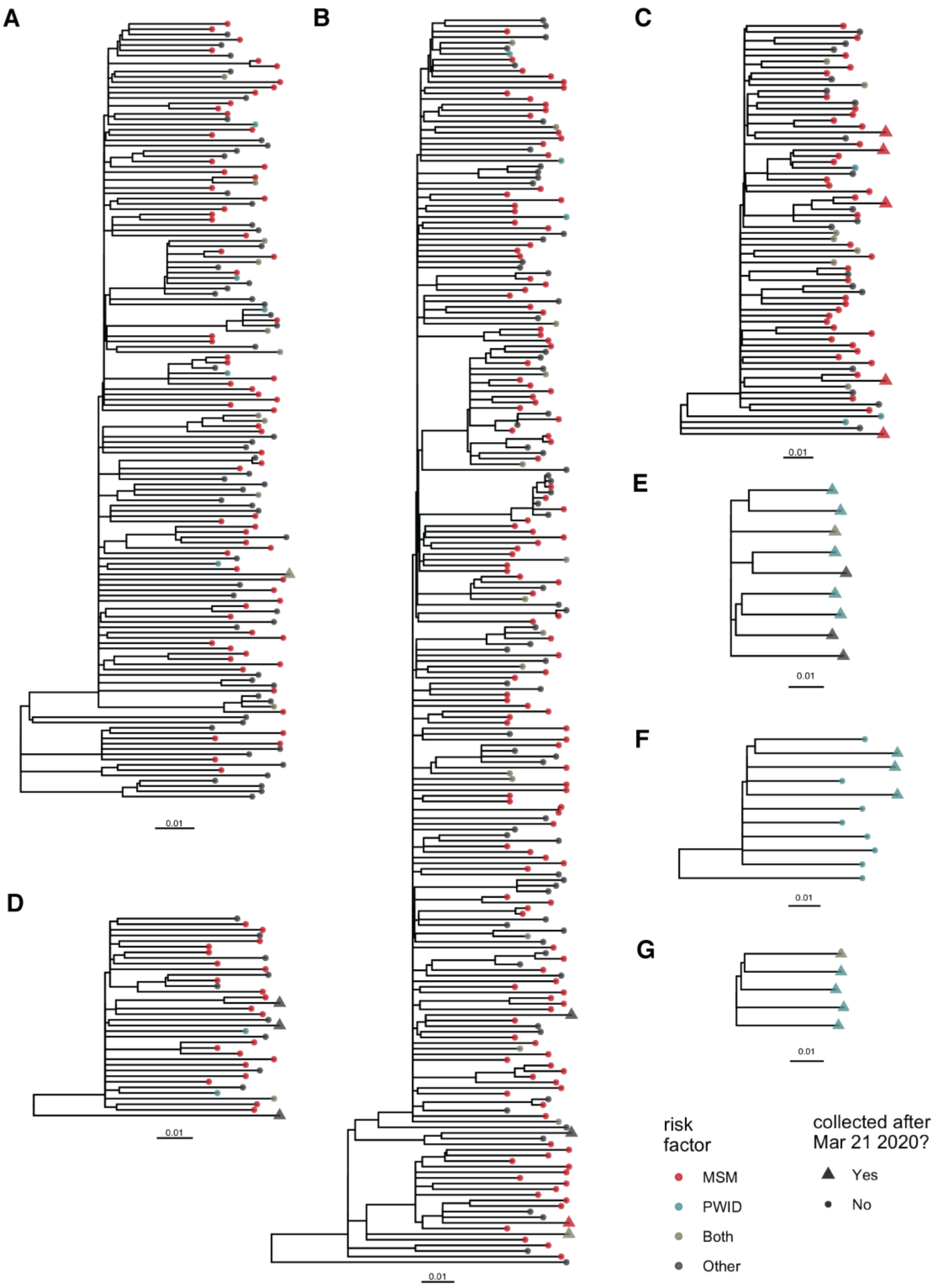

Figure S6. Phylogenetic trees for top-growing clusters. Phylogenetic trees representative of the clusters shown in Figure 4, pruned from the highest likelihood bootstrap tree. Panel labels correspond to clusters A-G. Tip shapes are coloured by reported risk factor. “Both” indicates individuals who reported both MSM and PWID as possible routes of transmission. “Other” indicates either individuals with no reported risk factor information available or individuals

who reported risk factors other than MSM or PWID, such as heterosexual sex, receipt of contaminated blood products or mother-to-child transmission. Sequences collected after March 21st, 2020 are marked by triangles. It is important to note that because our clustering algorithm relies on summarization of patristic distances derived from all bootstraps, branch lengths shown here are not directly representative of the distance values used to assign cluster membership.

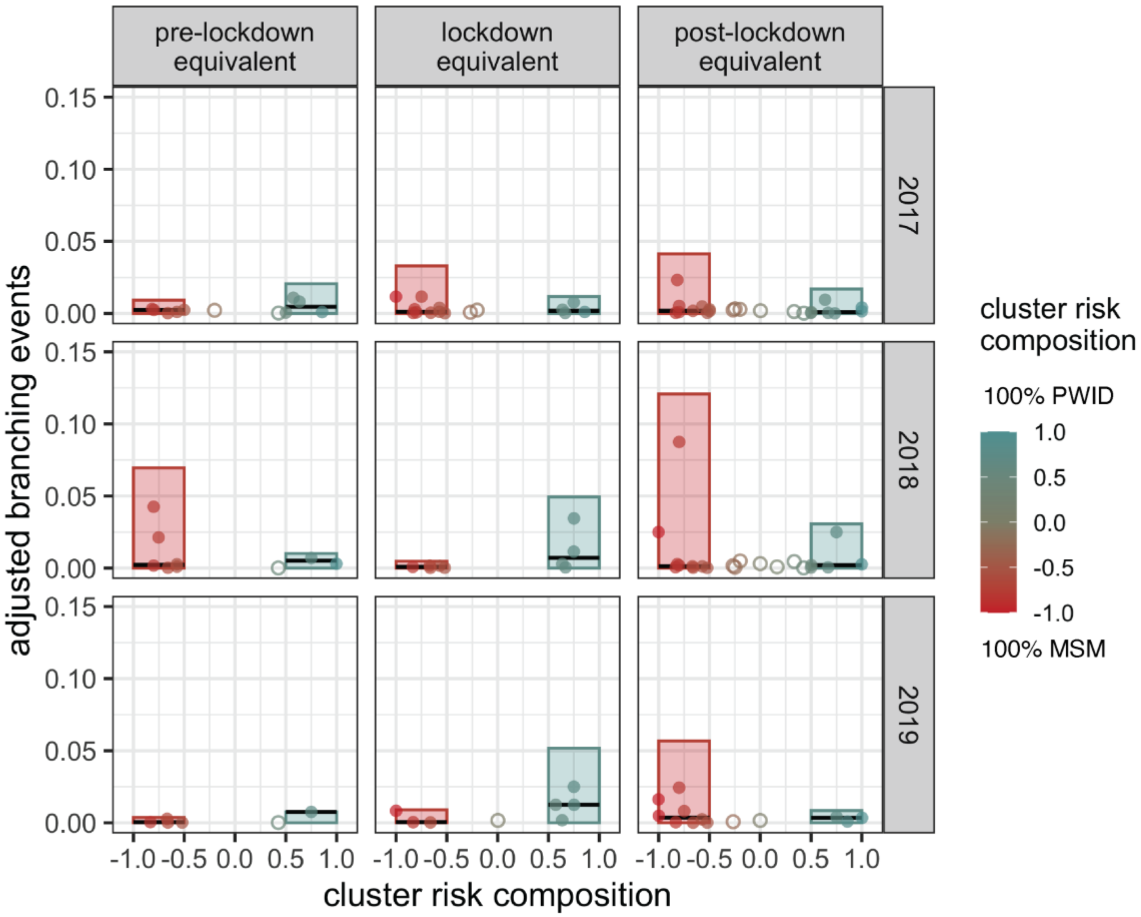

Figure S7. **Branching events in 2017 to 2019 by risk factor.** Bars represent the total number of daily median branching events inferred to be associated with clusters of each risk factor, normalized by cluster size and new diagnoses during a given time period. Observations were selected from periods between the same dates as the time periods studied in 2020, creating three "equivalent" time periods. In order to capture the branching events more likely to be associated with a certain risk factor, each event was assigned the risk factor composition of the cluster its descendants were members of. Following this, binary classification of cluster risk factors was done by labelling events assigned a risk composition of -0.5 or less as MSM and those with a risk composition of 0.5 or more as PWID. Ambiguous events assigned risk composition in between these thresholds were not included in the group totals. Closed circles represent clusters linked to events contributing to the risk group totals. Black horizontal lines represent the median of the closed circles associated with each risk group in each time period.

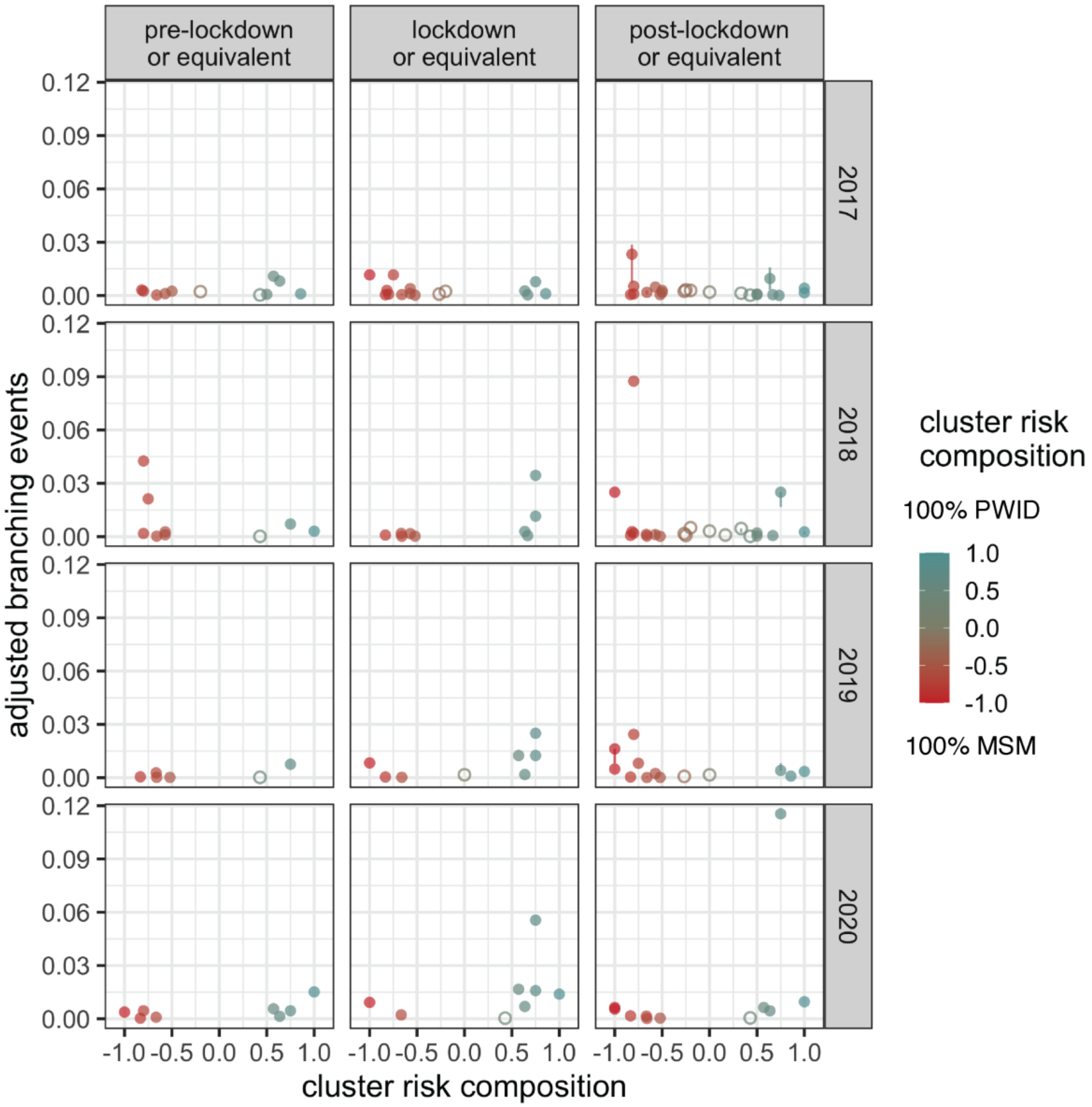

Figure S8. Variation in adjusted branching events by cluster across bootstraps. Each dot represents the median value for a cluster across bootstraps and the vertical lines show confidence intervals on these values. The absence of a confidence interval line indicates complete agreement across bootstraps. Clusters shown correspond to those in Figure 5 and S5. Each observation is coloured by cluster risk factor composition.

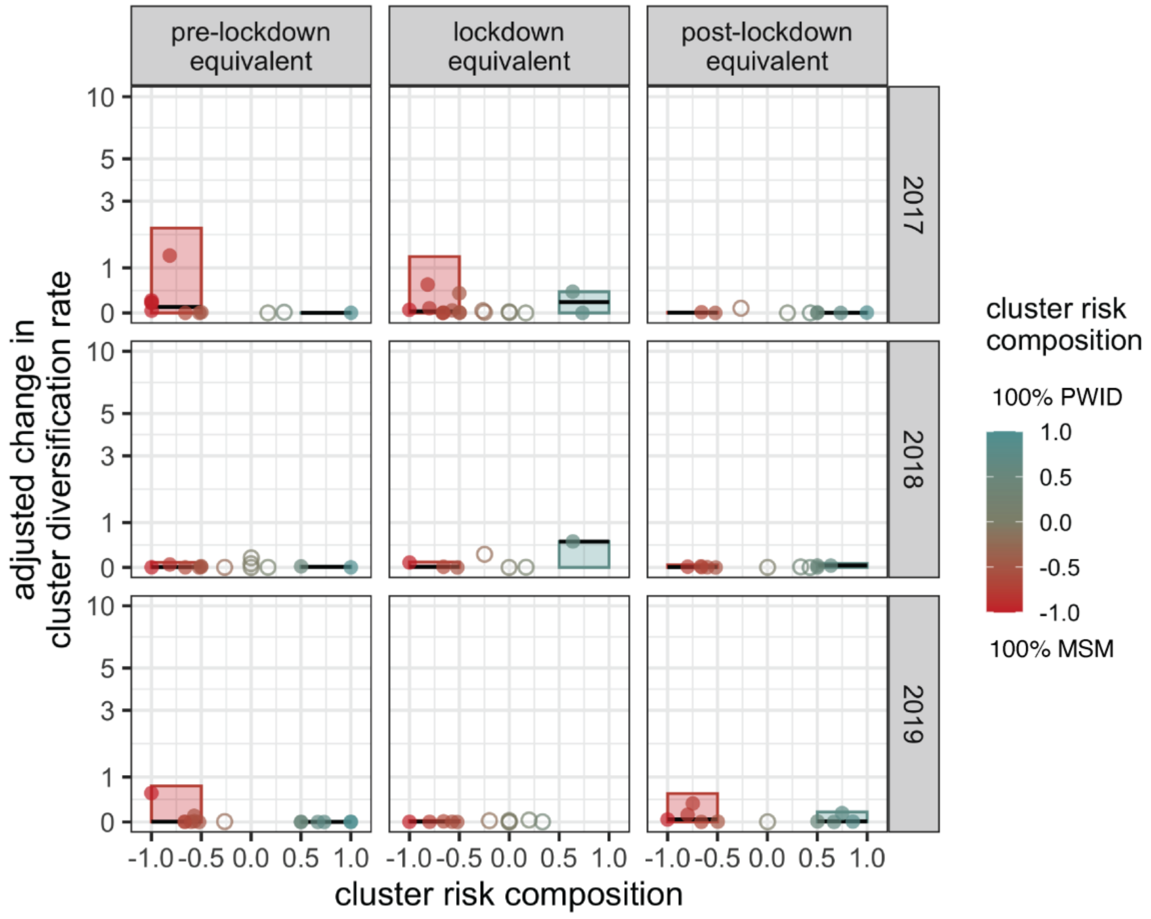

Figure S9. Cluster change in diversification rates within each time period in 2017 to 2019, by risk factor composition. Cluster median change in individual diversification rate between the beginning and end dates of a given time period, adjusted for cluster size and new diagnoses in that time period. Observations were selected from periods between the same dates as the time periods studied in 2020, creating three "equivalent" time periods. Binary classification of cluster risk factors was done by labelling clusters with a risk composition of -0.5 or less as MSM and those with a risk composition of 0.5 or more as PWID. Ambiguous clusters in between these thresholds were not included in the group totals. Closed circles represent clusters contributing to the risk group totals. Black horizontal lines represent the median of the closed circles associated with each risk group in each time period.

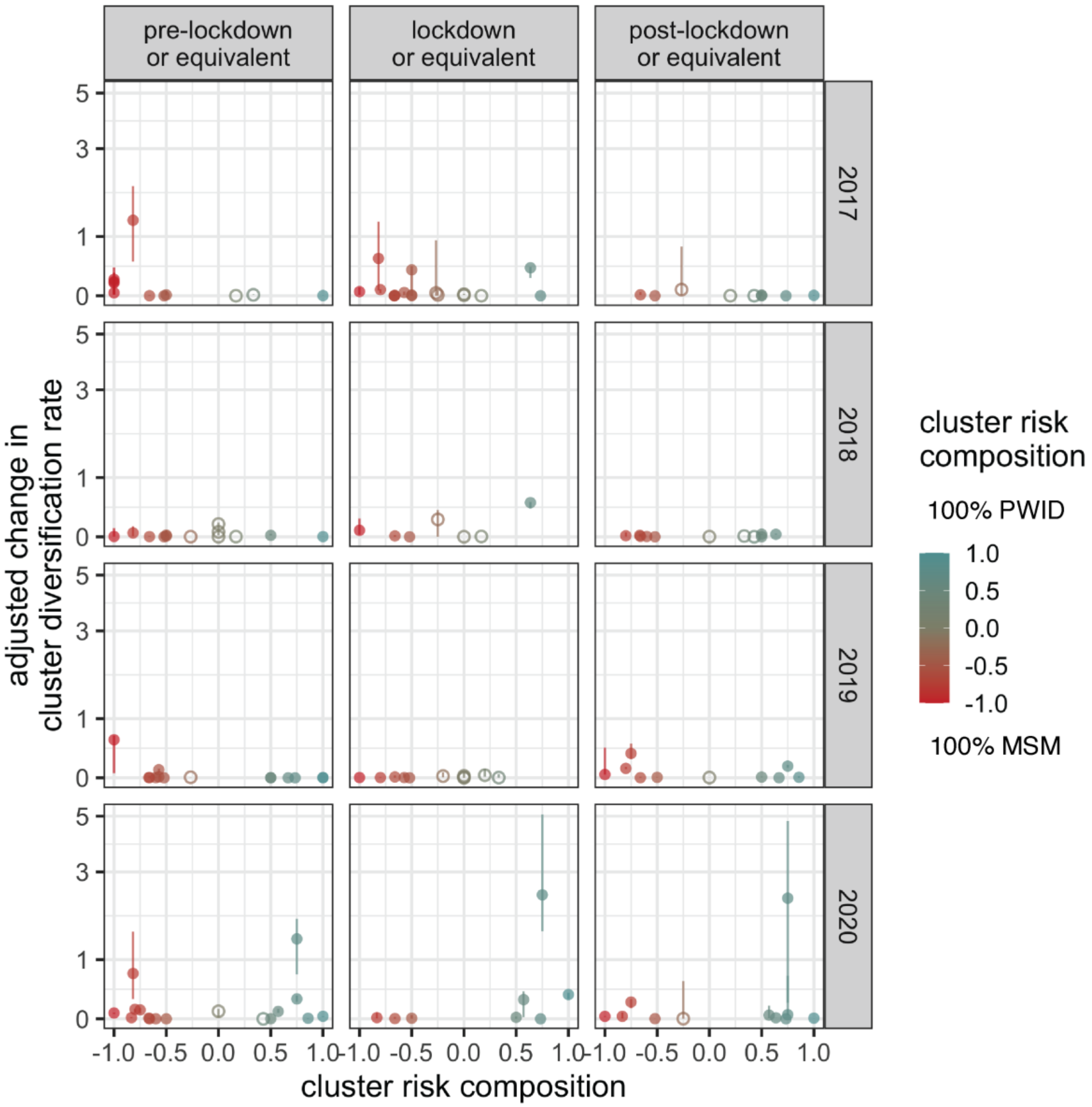

Figure S10. Variation in change in lineage-level diversification rate by cluster across bootstraps. Each dot represents the median value for a cluster across bootstraps and the vertical lines show confidence intervals on these values. The absence of a confidence interval line indicates complete agreement across bootstraps. Clusters shown correspond to those in Figure 5 and S9. Each observation is coloured by cluster risk factor composition.

Table S1. Cluster size during each 60-day time period in each year. Pre-lockdown (pre) includes January 22 – March 21, lockdown includes March 22 – May 20 and post-lockdown (post) includes May 21 – July 19. Only clusters that grew at some point following the implementation of restrictions are shown. Since the dataset only extends to June 4th, 2021, the 2021 post-lockdown period shown here notably only includes 15 days of sampling.

cluster	risk composition	cluster size in 2017			cluster size in 2018			cluster size in 2019			cluster size in 2020			cluster size in 2021		
		pre	lockdown	post	pre	lockdown	post	pre	lockdown	post	pre	lockdown	post	pre	lockdown	post
A	-0.52	120	120	121	131	132	133	138	140	140	142	142	144	147	147	147
B	-0.66	134	140	144	171	176	179	189	196	199	206	208	208	219	221	222
C	-0.83	58	58	58	59	59	59	59	59	59	65	66	69	69	70	70
D	-0.27	28	29	30	30	30	30	32	32	32	32	32	32	35	36	36
E	0.57											5	6	9	9	9
F	1													11	11	11
G	0.75														5	5
H	0.20	98	98	99	99	99	99	99	99	99	99	99	99	99	100	100
I	-1										6	6	6	8	8	8
J	0.75									6	9	9	10	10	10	11
K	1	19	19	19	19	19	19	21	21	21	21	21	22	22	22	22
L	-0.60	29	29	29	29	29	30	31	31	31	32	32	32	33	33	33
M	0.73	365	365	368	372	372	372	374	374	374	375	375	376	376	376	376
N	0	9	9	9	9	9	9	9	9	9	9	9	9	9	10	10
O	-0.75												5	6	6	6
P	-0.20	10	10	10	10	10	10	11	11	11	12	12	12	14	14	14
Q	-0.57	5	6	6	8	8	8	9	10	10	10	10	10	11	11	11
R	-0.67	50	52	52	52	52	52	52	52	52	53	53	53	53	54	54
S	-0.67	19	19	19	19	19	20	22	22	22	25	25	25	27	27	27
T	-0.8	9	11	11	12	12	13	16	16	16	17	17	17	19	19	19
U	-0.5	9	9	9	9	9	9	10	10	11	12	12	12	12	13	13
V	-0.25	35	36	36	37	38	38	39	39	39	39	39	40	40	40	40
W	-0.82	7	8	8	9	9	9	9	9	9	10	10	10	11	11	11
X	-1							5	5	5	5	5	7	7	7	7
Y	0.64	8	9	9	11	12	13	14	14	14	16	16	17	18	18	18
Z	0.17	25	25	25	27	28	28	28	28	28	28	28	28	29	29	29
AA	1	22	22	22	22	22	22	22	22	22	22	22	22	23	23	23
AB	0	13	13	13	13	13	13	13	13	13	13	13	13	13	14	14
AC	0.43	147	147	148	148	148	148	148	148	148	150	150	150	153	153	153
AD	0.86	23	23	23	23	23	23	25	25	25	27	28	28	28	29	30
AE	0.75										5	7	8	8	8	8
AF	0	11	11	11	11	11	11	11	11	11	11	11	11	11	11	12
AG	-1	7	7	7	7	7	7	7	7	7	7	7	7	8	8	8
AH	0.67	65	65	65	66	66	66	68	68	69	69	69	69	71	72	72
AI	0	22	22	22	22	22	22	22	22	22	22	22	22	23	23	23
AJ	0	15	15	15	15	15	15	15	15	15	16	16	16	17	17	17
AK	1	8	8	8	8	8	8	8	8	8	8	8	8	9	9	9
AL	-1	6	6	6	6	6	6	6	6	6	6	6	6	7	7	7
